# Hormonal Receptors in Skeletal Muscles of Dystrophic Mdx Mice

**DOI:** 10.1155/2013/604635

**Published:** 2012-12-27

**Authors:** David Feder, Ivan Rodrigues Barros Godoy, Maira Lazzarini Guimarães Pereira, Cledson Silveira Silva, Diego Nogueira Silvestre, Fernando Luiz Affonso Fonseca, Alzira Alves de Siqueira Carvalho, Rosangela Aparecida dos Santos, Maria Helena Catteli Carvalho

**Affiliations:** ^1^Department of Pharmacology, ABC Medical School, FMABC, Avenue Príncipe de Gales 821, 09060-650 Santo Andre, SP, Brazil; ^2^Department of Medicine, ABC Medical School, FMABC, Avenue Príncipe de Gales 821, 09060-650 Santo Andre, SP, Brazil; ^3^Federal University of São Paulo (UNIFESP), Rue Prof. Artur Riedel 270, 09972-270 Diadema, SP, Brazil; ^4^Department of Neurology, ABC Medical School, FMABC, Avenue Príncipe de Gales 821, 09060-650 Santo Andre, SP, Brazil; ^5^Department of Pharmacology, University of São Paulo (USP), Avenue Professor Lineu Prestes 152, 05508-900 São Paulo, SP, Brazil

## Abstract

*Introduction*. Several evidences show that muscles have an endocrine function. Glucocorticoid, estrogen, progesterone, and testosterone receptors have already been found in normal skeletal muscles, but not in dystrophic muscles. *Methods*. The gene expression of hormone receptors was compared between dystrophic and healthy muscles in mdx and C57BL6 mice strains. *Results*. The mdx mice showed a significant increase in the steroid receptors mRNA when compared to the C57BL6 mice: levels of androgen(s) receptors in the heart, estrogen receptors alpha in the EDL, and estrogen receptors beta in the quadriceps were increased. In addition, significant lowered levels of some other hormone receptors were found: corticosteroid receptors in the EDL and estrogen receptors alpha in the quadriceps. *Conclusion*. Dystrophic muscles bear significant differences in the expression of hormone receptors when compared to the C57BL6 mice strain. The importance of such differences is yet to be better understood.

## 1. Introduction

Duchenne muscular dystrophy (DMD) is an X-linked genetic disorder resulting in a defect in the muscle membrane protein called dystrophin. In the literature, there are more than thirty catalogued types of dystrophies. However, DMD is the most common kind, which affects 1 in every 3,500 male births. The disease initially manifests itself by muscular strength alterations, making the individual progressively lose the ability to walk and finally causing a reduction of the cardiac function and the respiratory muscles strength [1].

The most widely used experimental model for studying the disease is the mdx mouse. This kind of mouse bears a point mutation in the dystrophin gene. The mdx mutation occurred spontaneously due to a premature stop codon resulting in a termination in exon 23 of the dystrophin gene. The absence of dystrophin in mice produces a variety of phenotypes, higher than that observed in humans. Under normal conditions, the mdx mice show very few symptoms of the disease, and when subjected to intensive exercises, they present aggravation of the pathological alterations [2].

Corticosteroids are the only recommended treatment for DMD. The drug increases muscle strength and slows the onset of complications [1]. As muscles have an endocrine function and due to the fact that this disease causes muscle degeneration, the study of hormone receptors in these organs are of paramount importance. The mechanism of action of corticosteroids in DMD has not been fully elucidated [3].

Several evidences demonstrate that muscles have an endocrine function. Therefore, the study of hormone receptors in muscles has shown to be important. These hormones have an important role in the protein synthesis and degradation, in the increase of muscle strength, and in the muscle protection against the damage caused by exercises, facts that make relevant the quantification of hormone receptors in dystrophic muscles.

 The aim of this study was to evaluate the expression of hormone receptors in dystrophic muscles in mdx mice, comparing it with the expression of receptors in healthy muscles in C57BL6 mice.

## 2. Material and Methods

All procedures used in this study were approved and performed in accordance with the guidelines of the Animal Ethic Committee of FMABC.

Ten four-month-old mice, being five mdx and five C57BL6 strains, were studied. The latter was classified as control group. The animals were kept in spontaneous activity, with food and water ad libitum, and later sacrificed in a CO_2_ chamber. Some muscles, like the cardiac, the gastrocnemius, the diaphragm, the quadriceps, the soleus, and the extensorum digitalis longus (EDL), from both mice strains were dissected, frozen in liquid nitrogen, and stored at −80°C.

Total tissue RNA was extracted using Trizol Reagent (Invitrogen Co., USA), according to the manufacturer's instructions, quantified by absorbance at 260 nm, and stored in diethylpyrocarbonate-treated water at −80°C. The integrity of RNA was verified by agarose gel electrophoresis. Total RNA (2 ug) was used for first-strand cDNA synthesis by reverse transcriptase. MMLV and RNaseOUT were also added to protect the RNA during the process. 

An aliquot of the reaction was then submitted to PCR amplification with the appropriate primers. Alpha-actin was used as an internal control for the coamplification. The conditions for PCR were as follows: initial denaturation at 94°C for 5 minutes, followed by cycles of 94°C for 45 seconds, annealing of the temperature for 45 seconds, and 72°C for 1 minute. The final extension step occurs at 72°C for 10 minutes. The primers sequence(s), size of the PCR products, the annealing temperature, the number of cycles, the accession numbers of the target sequences, and the reactions conditions are presented in Table 1.

 Three pooled RNA aliquots were omitted from the reverse transcriptase reaction to ensure the absence of products other than those originated from the reverse-transcribed mRNAs. PCR products were loaded in 1% or 2% agarose gel electrophoresis, stained with 0.2 ug/mL ethidium bromide. The gel was subjected to ultraviolet light and photographed. The bands intensity was quantified by video densitometry and the signals were expressed in relation to the intensity of the alpha-actin amplicon in each coamplified sample. All primers and enzymes used in this protocol were purchased from Invitrogen Co. The results were subjected to analysis of variance, using the program GBStat and considering the level of significance *P* < 0.05.

## 3. Results

The results of the mRNA of the mice were placed in Table 2.

 Mdx mice showed significant raise in the expression of steroidal receptors mRNA when compared to the C57BL6 mice: androgen receptors in the heart, estrogen receptors alpha in the EDL, and estrogen receptors beta in the quadriceps were increased. In addition, significant lowered levels of some other hormone receptors were found: corticosteroid receptors in the EDL and estrogen receptors alpha in the quadriceps.

## 4. Discussion

 Muscles express steroid receptors that belong to the superfamily of intracellular receptors which control gene transcription. The effect is to induce or repress particular genes [4–6].

The biological effects of corticosteroids are mediated by glucocorticoid receptors [6]. These effects include control of salt/water homeostasis, blood pressure regulation, metabolic alterations, and cellular immunity [5]. Glucocorticoids have catabolic effect on skeletal muscles and, in high doses, they can cause a steroid myopathy [6]. The muscles most affected by corticosteroids are the quadriceps and the other muscles of the pelvic girdle, being type IIb fibers the most susceptible to this effect. Exercises are effective in preventing steroid myopathy [7]. 

The catabolic effects of glucocorticoids are mediated by numerous mechanisms. Glucocorticoids inhibit glucose uptake in skeletal muscles and may contribute to the degradation of muscle proteins. They may also act directly to inhibit the protein synthesis or increase its degradation. One of the genes responsible for this effect encodes the glutamine synthetase, an enzyme responsible for catalyzing the formation of glutamine that is exported from the muscle in catabolic conditions. In muscular atrophy induced by glucocorticoids, the efflux of glutamine is 25–30% of the total exported protein of the muscle. In rats, an increase of glutamine synthetase and the level of mRNA were observed after the administration of glucocorticoids. Another effect of glucocorticoids is the activation of genes responsible for the ubiquitin-proteasome system, increasing the proteolytic activity in the muscle. It is clear that the transactivation of many genes must be involved in muscle atrophy by corticosteroids. The increase of myostatin, a well-known protein inhibitor of muscle mass, has also been observed with the use of glucocorticoids [6, 8].

 Androgens, unlike glucocorticoids, exert an anabolic effect on muscles. The anabolic effects of androgens in skeletal muscles have been a source of controversy for more than six decades. Testosterone supplementation increases muscle mass in men; this effect is associated with hypertrophy of fibers type I and II with an increase of satellite cells and myonuclei. The mechanism by which testosterone increases muscle mass is poorly understood. Although the expression of androgen receptors have been described in muscle cells, it is not clear which cells express this receptor and what the target of its action is [9]. Recent studies have shown that androgens promote the differentiation of lineages of mesenchymal pluripotent cells into myogenic cells and inhibit the differentiation into cell lineages of adipose tissue. This effect is blocked by androgen receptor antagonists, indicating that this effect may be mediated through these receptors [9]. Testosterone receptor expression is observed in almost all satellite cells and in 50% of myonuclei [9]. Physical activity also plays a role in the expression of the androgen receptor. In rats subjected to prolonged and intense physical activity, there was an increase of the expression of androgen receptors in the soleus, but not in the EDL [10]. 

Estrogens also play an important role in skeletal muscles, regulating lipid and carbohydrate metabolism and acting on the muscle growth and strength [11–14].

Alpha and beta receptors are expressed in skeletal muscles in both men and women [15]. Many studies suggest that estrogens increase muscle strength, and variations in the voluntary muscle strength were observed in humans during the menstrual cycle [13, 14]. In other studies, there was no change in the muscle function with increased estrogen levels or fluctuations during the menstrual cycle [16, 17]. In female rats, the eccentric muscle contractions cause less histological changes than in male rats [18]. In ovariectomized rats, there is a greater expression of proteins related to stress and a greater degree of injury compared to normal female rats. This difference disappears upon cessation of estrogen treatment [19, 20]. Intense and prolonged physical activity increases the expression of alpha receptor mRNA in the gastrocnemius muscle in female rats, but not in male rats [21]. The expression of estrogen receptors alpha and beta is increased in men undergoing physical exercises. The mRNA of estrogen receptors alpha and beta is increased in highly trained men than in moderately trained ones, and that expression is correlated with muscle oxidative capacity. Estrogen receptors can be regulated by physical activities and may be involved in the process of adaptation to physical exercises [22]. Estrogen receptor alpha mRNA expression is higher in the soleus than in the gastrocnemius or the EDL [23]. 

There are few studies of hormone receptors and hormone effects in dystrophic muscles. 

Dubois and Almon [24] described a 100% increase in corticosteroid receptors in the leg muscles of mice with muscular dystrophy in relation to the control group; this increase was also found in chickens with muscular dystrophy [25].

The use of anabolic androgens was also tested in muscular dystrophy. DMD patients treated with oxandrolone were compared with a placebo group [26]; although patients treated with oxandrolone did not experience the loss of strength of the control group, the difference between both groups was not significant. However, the average QMT (quantitative muscle testing of four muscles) showed a significant increase in patients treated with oxandrolone compared with those treated with placebo. Besides the great anabolic power of oxandrolone in DMD, the drug promotes increased expression of genes that might partly explain its effect [27]. Skeletal muscles of mice with muscular dystrophy have testosterone receptors with the same characteristics of activity and affinity as those of normal mice.

In DMD patients, estrogen levels were higher than a control group matched by age. LH levels were normal and FSH levels were low [28]. The use of estrogens has already been evaluated in DMD. In eleven DMD patients treated with diethylstilbestrol, a reduction in the levels of CK and LDH was observed with no analysis of the muscular strength parameters [29]. Another study showed anabolic effect of diethylstilbestrol in 7 DMD patients [30]. Young female mdx mice show less myonecrosis and more fiber regeneration; mdx ovariectomized females show a reduction of fiber regeneration. Older mdx females present extensive fibrosis, increase of the permeability of the sarcolemma, and marked deposition of extracellular matrix components compared to mdx males [31]. This increased regeneration of mdx females at younger ages may be explained by a mitogenic effect of estrogen that binds to nuclear receptors. This effect has been confirmed in vivo with estradiol activating the proliferation of muscle satellite cells [32]. In spite of the fact that myoblasts from mice express the estrogen receptor in vitro, estradiol does not influence the proliferation of myoblasts [32]. It is suggested that estrogen would act indirectly, by inducing growth factors like IGF-1 and IL-6 [31, 32]. In our study, we observed that the expression of glucocorticoid receptors is reduced in the EDL muscle of dystrophic mice. As the treatment with glucocorticoids increases the expression of these receptors, we could speculate that this would be one of the probable mechanisms of action of corticosteroids. We also note that the expression of androgen receptors is reduced in the quadriceps and the gastrocnemius of mice with a mutation in the dystrophin gene. Since the expression of these receptors increases with testosterone administration, this would also be a likely benefic mechanism of treatment with androgens in muscular dystrophy.

 Another observation from our study was that the expression of estrogen receptors was increased in the EDL (estrogen alpha) and the quadriceps (estrogen beta) of dystrophic mice. This effect may explain the worst outcome of female mdx mice, which present extensive fibrosis, increased permeability of the sarcolemma, and marked deposition of extracellular matrix components, once they had been suffering a prolonged action of estrogens [33].

 Another fact found, which requires more studies, was the reduction in progesterone receptor expression in the diaphragm of dystrophic mice compared with healthy mice.

## 5. Conclusion

 This is a preliminary study and it represents the first study of hormone receptors in differentmuscles of mdx mice. The analysis of different muscle groups showed that dystrophic muscles have some significant differences in hormone receptor expression when compared to normal mice. Changes in expression of hormone receptors in dystrophic muscles reported here could explain some of the events related to the pathophysiology of DMD and the response to the therapy already described in the literature. Hence, carrying out more studies on the hormone receptor expression in DMD patients would open new path for the discovery of new drugs that might, more effectively, slow the disease progression and improve the quality of life of these patients.

## Figures and Tables

**Table 1:** RT-PCR analysis of various mRNAs: primes, size of the PCR products and conditions of the PCR reactions.

Gene	Primers sequence	Size of PCR product	Temperature (°C)	Number of cycles	Accession number at http://ncbi.nlm. nih.gov/
Androgen receptor	Sense: TACAACTTTCCGCTGGCTCT Antisense: CCGGAGTAGTTCTCCATCCA	464	58	30	NM013476.2

Glucocorticoid receptor	Sense: CGAGAGTCCTTGGAGGTCAG Antisense: GATCCTGCTGCTGAGAAAGG	410	55	24	X13358

Estrogen receptor Alpha	Sense: AATTCTGACAATCGACGCCAG Antisense: GTGCTTCAACATTCTCCCTCCTC	344	55	30 or 40*	NM007956

Estrogen receptor Beta	Sense: ACAGTCCTGCTGTGATGAAC Antisense: ACTAGTAACAGGGCTGGCAC	271	55	30 or 40*	U81451

Progesterone receptor	Sense: CCAGCATGTCGTCTGAGAAA Antisense: AAACACCATCAGGCTCATCC	426	60	40	NM008829

*α*-Actin	Sense: TGTGATGGTGGGAATGGGTCAG Antisense: TTGATGTCACGCACGATTTCC	513	55	28	AK151136

*Number of cycles varies with the saturation curve by the cycle of each tissue.

**Table 2:** Comparison of hormone receptor mRNA in different muscle groups studied in mdx mice and C57BL6 mice.

Receptor	Glucocorticoid		Androgen		Estrogen alpha		Estrogen beta		Progesterone	
	C57BL/6	mdx	C57BL/6	mdx	C57BL/6	mdx	C57BL/6	mdx	C57BL/6	mdx
Solium	1.49 ± 0.16	1.60 ± 0.89	0.99 ± 0.28	1.79 ± 0.83	1.86 ± 0.29	1.72 ± 0.64	1.15 ± 0.52	1.18 ± 0.46	0.79 ± 0.12	0.71 ± 0.43
EDL	1.90 ± 0.29	1.20 ± 0.16**	1.27 ± 0.79	1.34 ± 0.19	0.99 ± 0.18	1.40 ± 0.10**	0.78 ± 0.22	1.16 ± 0.26	#	#
Gastrocnemius	2.42 ± 0.68	2.87 ± 0.50	2.62 ± 0.34	2.21 ± 0.23*	4.34 ± 0.82	3.94 ± 0.54	1.00 ± 0.21	1.00 ± 0.17	#	#
Quadriceps	0.42 ± 0.10	0.35 ± 0.03	1.30 ± 0.28	0.89 ± 0.07*	1.52 ± 0.29	1.04 ± 0.12*	5.14 ± 0.92	9.17 ± 1.95*	#	#
Diaphragm	1.47 ± 0.16	1.25 ± 0.24	0.80 ± 0.33	0.91 ± 0.33	1.82 ± 0.49	1.25 ± 0.18	5.72 ± 1.60	4.11 ± 0.58	0.58 ± 0.16	0.34 ± 0.07*
Heart	1.37 ± 0.19	1.64 ± 0.35	0.39 ± 0.16	1.61 ± 0.52*	0.91 ± 0.34	1.57 ± 0.70	#	#	#	#

# nonexpression; **significance statistical.

## Abbreviations

cDNA: Complementary deoxyribonucleic acid
CK: Creatine kinase
DMD: Duchenne muscular dystrophy
EDL: Extensor digitorum longus
FSH: Follicle-stimulating hormone
IGF1: Insulin growth factor 1
IL-6: Interleukin-6
LDH: Lactase dehydrogenase
LH: Luteinizing hormone
MMLV: Moloney murine leukemia virus reverse transcriptase
mRNA: Messenger ribonucleic acid
PCR: Polymerase chain reaction
QTM: Quantitative muscle testing of four muscles
RNA: Ribonucleic acid
RNaseOUT: Ribonuclease inhibitor
RT: Reverse transcriptase.
